# Comparison of multiple linear regression and machine learning methods in predicting cognitive function in older Chinese type 2 diabetes patients

**DOI:** 10.1186/s12883-023-03507-w

**Published:** 2024-01-02

**Authors:** Chi-Hao Liu, Chung-Hsin Peng, Li-Ying Huang, Fang-Yu Chen, Chun-Heng Kuo, Chung-Ze Wu, Yu-Fang Cheng

**Affiliations:** 1 Department of Medicine, Division of Nephrology, Kaohsiung Armed Forces General Hospital, Kaohsiung, Taiwan, R.O.C.; 2 Department of Urology, Cardinal Tien Hospital, School of Medicine, Fu-Jen Catholic University, New Taipei City, Taiwan, R.O.C.; 3 Division of Endocrinology and Metabolism, Department of Internal Medicine, Department of Medical Education, Fu Jen Catholic University Hospital, School of Medicine, College of Medicine, Fu Jen Catholic University, New Taipei City, Taiwan, R.O.C.; 4 Department of Internal Medicine, Division of Endocrinology and Metabolism, Fu Jen Catholic University Hospital, New Taipei City, Taiwan, R.O.C.; 5 Department of Internal Medicine, Division of Endocrinology and Metabolism, Fu Jen Catholic University Hospital, School of Medicine, College of Medicine, Fu Jen Catholic University, New Taipei City, Taiwan, R.O.C.; 6 Department of Internal Medicine, Division of Endocrinology, Shuang Ho Hospital, New Taipei City, 23561 R.O.C.; 7 Division of Endocrinology and Metabolism, School of Medicine, College of Medicine, Taipei Medical University, Taipei, 11031 Taiwan, R.O.C.; 8 Department of Endocrinology and Metabolism, Changhua Christian Hospital, 135 Nanhsiao Street, Changhua City, 50006 Taiwan, R.O.C.; 9 Department of Medicine, Taipei Medical University, Taipei, Taiwan, R.O.C.

**Keywords:** Machine learning, Cognitive function, Type 2 diabetes

## Abstract

**Introduction:**

The prevalence of type 2 diabetes (T2D) has increased dramatically in recent decades, and there are increasing indications that dementia is related to T2D. Previous attempts to analyze such relationships principally relied on traditional multiple linear regression (MLR). However, recently developed machine learning methods (Mach-L) outperform MLR in capturing non-linear relationships. The present study applied four different Mach-L methods to analyze the relationships between risk factors and cognitive function in older T2D patients, seeking to compare the accuracy between MLR and Mach-L in predicting cognitive function and to rank the importance of risks factors for impaired cognitive function in T2D.

**Methods:**

We recruited older T2D between 60–95 years old without other major comorbidities. Demographic factors and biochemistry data were used as independent variables and cognitive function assessment (CFA) was conducted using the Montreal Cognitive Assessment as an independent variable. In addition to traditional MLR, we applied random forest (RF), stochastic gradient boosting (SGB), Naïve Byer’s classifier (NB) and eXtreme gradient boosting (XGBoost).

**Results:**

Totally, the test cohort consisted of 197 T2D (98 men and 99 women). Results showed that all ML methods outperformed MLR, with symmetric mean absolute percentage errors for MLR, RF, SGB, NB and XGBoost respectively of 0.61, 0.599, 0.606, 0.599 and 0.2139. Education level, age, frailty score, fasting plasma glucose and body mass index were identified as key factors in descending order of importance.

**Conclusion:**

In conclusion, our study demonstrated that RF, SGB, NB and XGBoost are more accurate than MLR for predicting CFA score, and identify education level, age, frailty score, fasting plasma glucose, body fat and body mass index as important risk factors in an older Chinese T2D cohort.

## Introduction

The prevalence of type 2 diabetes (T2D) has significantly increased in recent decades. As stated in the 2021 Diabetes Atlas published by the International Diabetes Federation, an estimated 537 million individuals are estimated to be living with diabetes worldwide [1]. The annual cost for providing care to these individuals has reached 966 billion US dollars, with a substantial portion allocated to treating microvascular and macrovascular diseases, common complications resulting from poorly managed blood glucose levels [2]. Approximately half of T2D patients succumb to cardiovascular diseases, including myocardial infarction and stroke [1]. Furthermore, T2D is linked to a higher risk of developing dementia, which has emerged as a prevalent public health concern in aging populations. Current consensus suggests that individuals with T2D have 1.43 to 1.46 times greater odds of developing dementia compared to those without diabetes [3–7].

The term "dementia" is defined as “the loss of cognitive functioning — thinking, remembering, and reasoning — to such an extent that it interferes with a person's daily life and activities” by the National Institute of Aging [8]. According to a 2021 report published by the World Health Organization (WHO), over 55 million individuals worldwide are currently affected by dementia, with nearly 10 million new cases being diagnosed annually [9]. Taiwan has followed a similar pattern, with a nationwide study indicating an 8.2% prevalence of dementia within the population. Currently, dementia stands as the seventh leading cause of death and significantly contributes to disability and dependency in the world [10]. Dementia can stem from a variety of neurodegenerative and non-neurodegenerative disorders. The most prevalent form of dementia is mixed dementia, characterized by a combination of Alzheimer's disease and cerebral vascular disease [11]. Risk factors for Alzheimer's disease that cannot be modified include age, female sex, Hispanic ethnicity, black race, and the presence of the apolipoprotein E gene [12]. Conversely, there are also modifiable risk factors. The INTERSTROKE study identified hypertension, T2D, diet (fruit and vegetables), high alcohol consumption, smoking, low levels of physical activity, high waist-hip ratio, psychosocial stress, and depression as examples of modifiable risk factors [13]. The underlying pathophysiology between T2D and dementia might be explained by the role of insulin resistance, which is one of the major causes for developing T2D. Evidence has shown that insulin resistance is found in the cortex and hippocampus [14]. Ho et al. showed that a high fat diet induced peripheral insulin resistance, reducing basal signaling in the cerebral cortex which in turn exacerbates the molecular pathology for Alzheimer disease in a genetic background [15]. Molecules such as PKB and GSK3 link T2M and dementia [16].

Machine learning (Mach-L) has been widely applied in medical research in recent years. Mach-L leverages recent advances in computational power and computer algorithms to autonomously achieve the objectives of many studies in medical research, as proposed by Mitchell et al. [17]. Mach-L has emerged as a compelling alternative to traditional multiple linear regression (MLR) for analyzing data [18–20] because of its ability to capture non-linear relationships and intricate interactions among numerous predictors without the assumption of a normal data distribution. As a result, Mach-L can potentially outperform conventional MLR in disease prediction [20]. However, in research on the association between T2D and dementia, Mach-L has predominantly been used for the diagnosis or prediction of dementia using imaging techniques [21, 22]. Only a few studies have used Mach-L to forecast dementia based on the aforementioned risk factors, particularly among patients with diabetes. Consequently, this study uses Mach-L as a comparative model with a two-fold objective: firstly, to assess whether Mach-L could surpass traditional MLR in predicting cognitive function assessment scores (CFA), and secondly, to compare the relative significance of the risk factors for CFA as determined by Mach-L in previous studies. According to Javeed’s review article, previous work can be categorized as voice, image and clinical variables modality [23]. Since the present study uses clinical variables, we only focus on this modality. Between 2011 to 2022, a total of 25 studies used Mach-L and clinical variables to predict dementia with between 4 and 350 variables. None of these studies focused on T2D patients. However, Chiu et al. used 45 variables, the most important of which included memory, orientation, judgement, community affairs and home hobbies, and producing an area under the receiver-operation characteristic of 0.94 [24]. Other studies used electrocardiogram, hand written drawings, or voice recordings for prediction [25–27]. The present study is the only one using demographic, biochemistry, lifestyle data for prediction.

We gathered data on cognitive function from Chinese older adults diagnosed with T2D. The CFA served as the independent variable (y), while demographic factors and biochemistry data were used as the dependent variables (x). Four distinct Mach-L methods were implemented: namely random forest (RF), stochastic gradient boosting (SGB), Naïve Bayes (NB), and eXtreme Gradient Boosting (XGBoost). Our primary aim was to assess whether Mach-L could outperform traditional MLR in predicting CFA, while also comparing the relative significance of risk factors determined by Mach-L against prior studies.

## Methods

### Participant enrollment

**Fig. 1:**
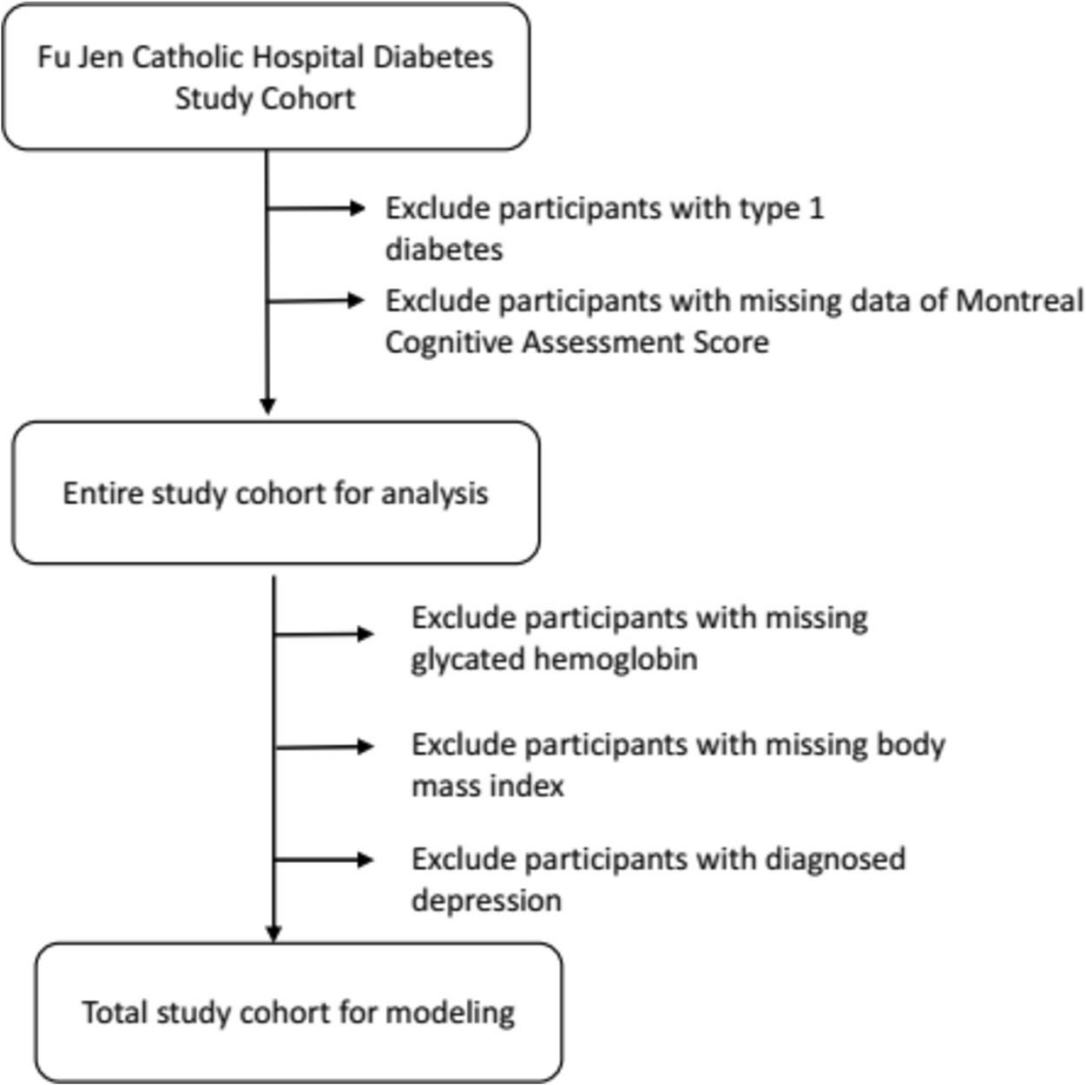
Flowchart of sample selection from the Fu Jen Catholic Hospital diabetes study cohort

Data for this study were derived from the diabetic outpatient clinic in Fu Jen Catholic Hospital in Taiwan from Jan to Dec 2022. The data were collected anonymously from the medical record database. The study protocol was approved by the institutional review board of the Fu Jen Catholic Hospital (FJUH111218). Since the data were retrieved from the electronic medical records and no sampling of the participants was needed, the protocol went through a short review, and the IRB waived consent requirements. Inclusion criteria were: 1. T2D. 2. Age between 60 to 95 years old. 3. Body mass between 22 to 30 kg/m^2^. 4. Glycated hemoglobin between 6.5 to 10.5%. Exclusion criteria were: 1. Type 1 diabetes. 2. Age under 50 or over 75. 3. BMI less than 22 or higher than 30 kg/m2. 4. Glycated hemoglobin less than 6.5% and higher than 10.5%. 5. Participants had not undergone the Montreal Cognitive Assessment at the time of the study. 6. Had a previous diagnosis of depression. 7. Were not under regular dialysis. The rationale we only enrolled patients between 60–95 was due to the high prevalence of dementia in this age group. Figure 1 illustrates the participant selection process.

### Data collection

On the day of the study, a senior nursing staff member recorded the participants’ medical history, including information on any current medications, and performed a physical examination. Participants’ marriage status, educational attainment, and smoking and drinking status were all collected at the same time. Waist circumference (WC) was measured horizontally at the level of the natural waist. BMI was calculated as the participants’ body weight (kg) divided by the square of the participants’ height (m). Both systolic blood pressure (SBP) and diastolic blood pressure (DBP) were measured by standard mercury sphygmomanometers on the right arm while seated. The Center for Epidemiologic Studies Depression Scale (CES-D) was used to evaluate depression status. The scale includes 20 questions, each with a score range from 0 – 3, where a higher total score indicates more severe depression [28]. The Fried Frailty Phenotype [29] was used to assess frailty. Participants were scored on five items, for which scores of 1–2 (inclusive) indicate pre-frailty, and over 3 (inclusive) is frailty. All the aforementioned data were regarded as independent variables. The Montreal cognitive assessment (MoCA)Taiwan version was used to assess cognitive function [30]. MoCA is because it is a widely used test and has been shown to have good sensitivity and specificity to detect participants with mild cognitive impairment [31]. The total score is 30 and ≧ 26 is regarded as no cognitive impairment. This is quantification of CFA and also a continuous and dependent variable of the present study.

After fasting for 10 h, blood samples were drawn for biochemical analyses. Plasma was separated from the blood within 1 h of collection and stored at 30 °C until analysis for fasting plasma glucose (FPG) and lipid profiles. FPG was measured using a glucose oxidase method (YSI 203 glucose analyzer, Yellow Springs Instruments, Yellow Springs, OH, USA). Total cholesterol and triglyceride (TG) levels were measured using a dry, multilayer analytical slide method with the Fuji Dri-Chem 3000 analyzer (Fujifilm, Tokyo, Japan). Serum high-density lipoprotein cholesterol (HDL-C) and low-density lipoprotein cholesterol (LDL-C) concentrations were analyzed using an enzymatic cholesterol assay, following dextran sulfate precipitation. A Beckman Coulter AU 5800 biochemical analyzer determined the urine microalbumin by turbidimetry. Finally, the creatinine level was measured by using a Beckman Coulter AU 5800 biochemical analyzer with the Kinetic Modified Jaffe method.

### Traditional statistics

The relationships between CFA and the other risk factors were assessed by Pearson’s correlation. All data are presented as mean ± standard deviation. *p* < 0.05 is considered statistically significant.

### Machine learning methods

As previously noted, the present study uses RF, SGB, NB and XGBoost to construct models to predict CFA score and to rank of importance of risk factors. These Mach-L methods have been used widely in healthcare applications and do not need prior assumptions regarding data distribution [32–41]. MLR was used as the benchmark for comparison.

Our previous article [32] provides detailed descriptions of these three methods. The Naïve Bayes (NB) Classifier (NB) is a popular Mach-L model used for classification tasks, able to sort objects according to specific characteristics and variables based on the Bayes theorem. It calculates the probability of hypotheses for presumed groups [33].

**Fig. 2:**
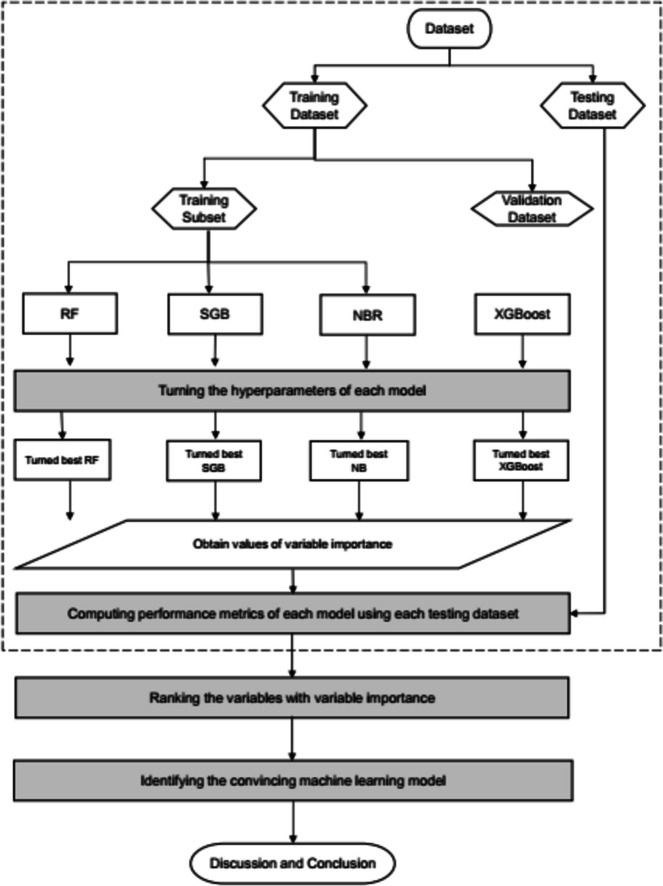
The flowchart of the proposed machine learning methods

The Mach-L method used here is adapted from Huang et al. [32]. The dataset was randomly divided into two subsets: 80% for training and 20% for testing. A tenfold cross-validation (CV) technique for hyperparameter turning was used (Fig. 2). According to the proposed scheme, for the development of effective RF, SGB, NB and XGBoost models we use tenfold cross-validation to tune and evaluate the hyperparameters of each method. The baseline MLR method without hyperparameter tuning was constructed using the proposed scheme. The values of hyperparameters which generate the best RF, SGB, NB and XGBoost models are listed in Table 1.

**Table 1:** Summary of the values of the hyperparameters for the best RF, SGB, NB and XGBoost models

Methods	Hyperparameters	Best Value	Meaning
RF	mtry	8	The number of random features used in each tree
	ntree	500	The number of trees in forest
XGBoost	nrounds	100	The number of tree model iterations
	max_depth	3	The maximum depth of a tree
	eta	0.4	Shrinkage coefficient of tree
	gamma	0	The minimum loss reduction
	subsample	0.75	Subsample ratio of columns when building each tree
	colsample_bytree	0.8	Subsample ratio of columns when constructing each tree
	rate_drop	0.5	Rate of trees dropped
	skip_drop	0.05	Probability of skipping the dropout procedure during a boosting iteration
	min_child_weight	1	The minimum sum of instance weight
NB	fL	0	Adjustment of Laplace smoother
	usekernel	TRUE	Using kernel density estimate for continuous variable versus a Gaussian density estimate
	adjust	1	Adjust the bandwidth of the kernel density
SGB	n.trees	50	The number of tree model iterations
	interaction.depth	1	The iterations depth of a tree
	shrinkage	0.1	Subsample ratio of columns when building each tree
	n.minobsinnode	10	The minimum number of instances per leaf Node

*RF* Random forest, *SGB* Stochastic gradient boosting, *NB* Naïve Byer’s classifier, *XGBoost* eXtreme gradient boosting

Some of the variables in this study are numerical, thus the metrics used for model performance comparison are the mean absolute percentage error (MAPE), symmetric MAPE (SMAPE), relative absolute error (RAE), root relative squared error (RRSE) and root mean square error (RMSE). The calculation of these model error metrics is shown in Table 2. R software version 4.0.5 and RStudio version 1.1.453 with the required packages installed were used.

**Table 2:** Equation of performance evaluation metrics

Metrics	Description	Calculation
SMAPE	Symmetric Mean Absolute Percentage Error	$SMAPE=\frac{1}{n}\sum_{i=1}^{n}\frac{\left|{y}_{i}-{\widehat{y}}_{i}\right|}{\left(\left|{y}_{i}\right|+\left|{\widehat{y}}_{i}\right|\right)/2}\times 100$
MAPE	Mean Absolute Percentage Error	$MAPE=\frac{1}{n}\sum_{i=1}^{n}\left|\frac{{y}_{i}-{\widehat{y}}_{i}}{{y}_{i}}\right|\times 100$
RAE	Relative Absolute Error	$RAE=\sqrt{\frac{{\sum }_{i=1}^{n}{\left({y}_{i}-{\widehat{y}}_{i}\right)}^{2}}{{\sum }_{i=1}^{n}{\left({y}_{i}\right)}^{2}}}$
RRSE	Root Relative Squared Error	$RRSE=\sqrt{\frac{\sum_{i=1}^{n}{\left({y}_{i}-{\widehat{y}}_{i}\right)}^{2}}{\sum_{i=1}^{n}{\left({y}_{i}-{\widehat{y}}_{i}\right)}^{2}}}$
RMSE	Root Mean Squared Error	$RMSE=\sqrt{\frac{1}{n}\sum_{i=1}^{n}{\left({y}_{i}-{\widehat{y}}_{i}\right)}^{2}}$

where ${\widehat{y}}_{i}$ and ${y}_{i}$ represent predicted and actual values, respectively; $n$ stands the number of instances

## Results

### General description of the study cohort

Totally, there were 580 participants were enrolled. Due to different causes that did not meet our inclusion criteria, only 197 participants were remained for analysis (women: 98, men: 99) (Fig. 1). We recruited older adults with T2D aged between 60 to 95 years old. The reason for this age range was because that they had a higher chance to have deteriorated CFA. The mean age was 73.0 ± 6.0 y/o with a mean BMI of 25.8 ± 3.9 kg/m^2^. In terms of demographics, 71.43% (140 participants) of respondents were currently married, 93.97% (191 participants) had an education level between elementary school and college, 27.55% (54 participants) were smokers and 25.51% (50 participants) consumed alcohol on a regular basis. Table 3 summarizes all descriptive characteristics.

**Table 3:** Participant percentage and mean (± standard deviation) of the participants’ demographic data and risk factors

Characteristics	Mean ± SD
N number (male/female)	196 (99/97)
Age	73.0 ± 6.0
Body mass index	25.8 ± 3.9
Systolic blood pressure	137.4 ± 18.4
Diastolic blood pressure	72.5 ± 11.2
Body fat percentage	32.0 ± 7.7
Fasting plasma glucose	142.5 ± 37.6
Alanine aminotransferase	24.3 ± 10.9
Triglyceride	117.3 ± 56.3
Low density lipoprotein cholesterol	91.6 ± 28.2
High density lipoprotein cholesterol	52.3 ± 15.8
Glycated hemoglobin	7.5 ± 1.3
Frailty total score	3.3 ± 1.7
Montreal cognitive assessment score	25.2 ± 4.6
Marriage	n (%)
Unmarried	8 (4.08%)
married living together	140 (71.43%)
married living apart	4 (2.04%)
divorced	8 (4.08%)
widowed	36 (18.37%)
Education	n (%)
Illiteracy	5 (2.55%)
elementary school	50 (25.51%)
junior high school	25 (12.76%)
senior high school	48 (24.49%)
College	60 (30.61%)
Graduate school	7 (3.57%)
Doctor degree	1 (0.51%)
Smoking	54 (27.55%)
Alcohol	50 (25.51%)

Marriage status: Unmarried: 0, married living together: 1, married living apart: 2, divorced: 3, widowed: 4; Education: Illiteracy: 0, elementary school: 1, junior high school: 2, senior high school: 3, college: 4, Graduate school: 5, Doctor degree: 6; The Montreal cognitive assessment Taiwan version was used to assess cognitive function; Cognitive function assessment score was conducted using the Montreal Cognitive Assessment. The evaluation items are visuospatial/executive, naming, memory, attention, language, abstraction, delayed recall and orientation

The details and mean (± standard deviation) of all the risk factors are shown in Table 3.

### Results of simple correlation between CFA score and other variables

Table 4 shows that smokers and alcohol consumers had higher CFA scores. Next, we used Pearson’s correlation on variables assessed and found that age, education, and frailty were all positively correlated with CFA, while body fat was negatively correlated (Table 5). In descending level of significance, the most highly correlated factors are education level, age, frailty status and body fat.

**Table 4:** The cognitive function assessment score in smoker, drinker and non-smoker and non-drinker

	Yes (percentage of participants)	No (percentage of participants)
Smoking status	3.74 ± 1.6 (27.55%)	3.14 ± 1.57 (72.45%)
Drinking status	3.88 ± 1.78 (25.51%)	3.10 ± 1.58 (74.49%)

Cognitive function assessment score was conducted using the Montreal Cognitive Assessment. The evaluation items are visuospatial/executive, naming, memory, attention, language, abstraction, delayed recall and orientation

**Table 5:** Relationships between cognitive function assessment score and other risk factors

Related variables	Age	Education	Frailty	Body fat	BMI	SBP	ALT	LDL-C	TG	FPG	HDL-C	DBP	HbA1c
Cognition	0.273***	0.443***	0.243***	-0.209***	-0.109	0.018	0.094	0.088	0.057	-0.020	-0.019	-0.105	0.010

*BMI* Body mass index, *HDL-C* High density lipoprotein cholesterol, *LDL-C* Low density lipoprotein cholesterol, *SBP* Systolic blood pressure, *DBP* Diastolic blood pressure, *ALT* Alanine aminotransferase, *FPG* Fasting plasma glucose, p: * < 0.05, ** < 0.01,*** < 0.005. Cognitive function assessment score was conducted using the Montreal Cognitive Assessment. The evaluation items are visuospatial/executive, naming, memory, attention, language, abstraction, delayed recall and orientation

### Accuracy comparison between MLR and four machine learning methods

Table 6 compares model performance for MLR, RF, SGB, NB and XGBoost. The MAPE, SMAPE, RAE, RRSE and RMSE values of RF, SGB and XGBoost were all smaller than those of the MLR, except for NB. This indicates that RF, SGB and XGBoost are more accurate than MLR. Taking MAPE for example, the MAPE of MLR was 0.61, higher than for RF, SGB, NB and XGBoost. Similar trends could also be noted in the other three error types. These findings strongly indicate that Mach-L method outperform MLR.

**Table 6:** Comparison with MAPE, SMAPE, RAE, RRSE and RMSE between Linear and machine learning methods

	MAPE	SMAPE	RRSE	RMSE
Linear	0.61	0.135	0.855	4.172
RF	0.599	0.131	0.851	4.153
SGB	0.606	0.126	0.852	4.159
NB	0.599	0.124	0.82	4.003
XGBoost	0.439	0.113	0.697	3.403

Data showed as mean; *RF* Random forest, *SGB* Stochastic gradient boosting, *NB* Naïve Bayes classifier, *XGBoost* eXtreme gradient boosting, *MAPE* Mean absolute percentage error, *SMAPE* Symmetric MAPE, *RAE* Relative absolute error, *RRSE* Root relative squared error, *RMSE* Root mean square error. The errors were used to compare the accuracies of the models. The smaller the errors, the better the model was

### Variable importance derived from the four Mach-L methods

**Fig. 3:**
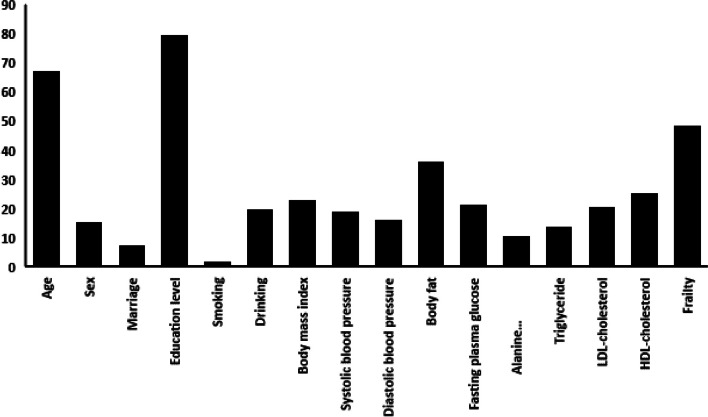
The percentage of importance of the risk factors. The Fried Frailty Phenotype: Participants were scored on five items, for which scores of 1–2 (inclusive) indicate pre-frailty, and over 3 (inclusive) is frailty

Table 7 presents the average ranks the four Mach-L methods, where the darker blue color indicates greater impact on CFA score. Education level is ranked highest by all four Mach-L methods, followed by age, except for NB which ranked age third, for an average of 2.25. Similarly, NB ranked frailty fourth, while the other three methods ranked it third, with an average of 3.25. All four methods consistently ranked body fat, BMI and FPG respectively in fourth through sixth. The rank of the importance is given in Table 7. In the same time in order to show their relative importance between variables, Fig. 3 is given. The original values of the percentage of importance are displayed.

**Table 7:**
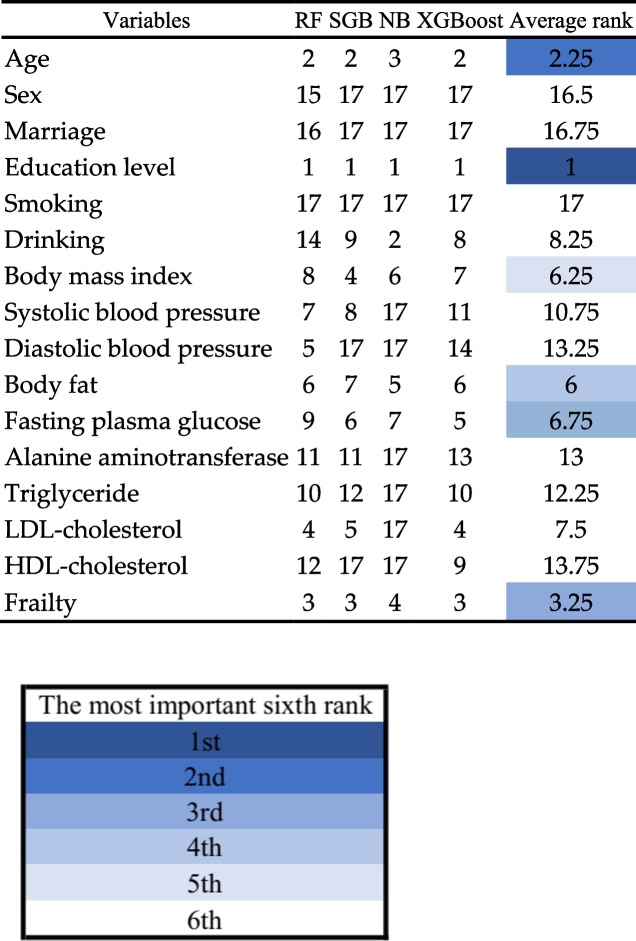
The ranks of the importance of risk factors derived from multiple linear regression, random forest and extreme gradient boost

The Fried Frailty Phenotype: Participants were scored on five items, for which scores of 1–2 (inclusive) indicate pre-frailty, and over 3 (inclusive) is frailty

**Fig. 4:**
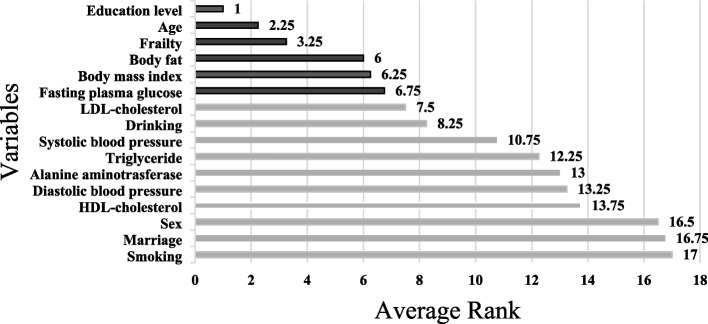
The ranks of the risk factors derived from three different machine learning methods. The unit of age is year; the education levels were classified as the following: illiteracy, elementary, junior, senior, college, graduate school and doctor degree. The Fried Frailty Phenotype: Participants were scored on five items, for which scores of 1–2 (inclusive) indicate pre-frailty, and over 3 (inclusive) is frailty

However, since these ranks are not in the order from the most to least important, Fig. 4 provides a graphical presentation that clearly shows the most important risk factors are education level, age, frailty score, FPG, body fat and BMI.

## Discussion

### Highlight of the study

Among the four different Mach-L methods, RF, SGB, and XGBoost outperformed MLR, identifying education level, age, frailty score, FPG and BMI as the key risk factors for detecting abnormal CFA scores, in descending order of importance.

Mach-L methods have several common characteristics: 1. They do not need hypotheses or assumptions such as normally distributed data sets. 2. They can capture non-linear relationships better than MLR. 3. They can iterate until the best fitting model is obtained. While Mach-L methods have been equated to a ‘black box’, in that their internal operations are not easily perceived, they do outperform MLR in terms of error frequency.

### Relationships between education level and CFA score

Our results show that education level is the most important risk factor for CFA, with lower scores significantly associated with lower educational attainment, a finding in line with most major studies. For example, the PAQUID project followed 3675 non-dementia participants for 5 years, finding that the hazard ratio for dementia in no-education and primary-school education participants had significantly higher risk for developing dementia (respectively 1.83 and 1.49 times greater risk their more educated counterparts) [37]. A 6-year longitudinal study in Japan of 51,186 individuals from 346 communities found that low community-level educational attainment was also associated with higher incidence of dementia [38]. At present, it is generally agreed that this positive relationship between cognitive function and education level can be explained by the fact that those with lower education typically have less physical and social resources within their communities. Moreover, low educational level is also related to relatively unhealthy lifestyles and lack of immediate health support or bonding social capital [39]. These are all the plausible underlying causes to explain this relationship.

### Relationship between age and CFA score

Consistent with other major studies, age is found to be the second important factor related to CFA score, as aging can cause brain degeneration and injury [40]. The Rotterdam Study of 7,046 participants found that the incidence of dementia increased from 0.6 to 97.2 per 1,000 person-years from the youngest to the oldest 5-year age category [41]. A meta-analysis of 13 studies prepared by Gao et al., also found that dementia increased with age [42]. However, it is important to note that the underlying causes of poor cognitive function are different in younger and older persons. For younger people, the main pathological feature of dementia is more typically related to neocortical neuritis plaques, as opposed to cerebral atrophy for those aged over 95 [43].

### Relationship between frailty score and CFA score

Frailty score was found to be the third most important factor for CFA. It is generally recognized that both physical and cognitive function decrease with age. In a cohort of 5,038 participants aged ≥ 55, Szlejf et al., found a negative relationship between sarcopenia and cognitive function (β = -0.20, 95% confidence interval = -0.38; -0.01, *p* = 0.03) after adjusting for other confounding factors [44]. While their study is cross-sectional, it still provides important evidence given the inclusion of middle-age adults. However, their use of a categorical analysis is less persuasive than a continuous variable analysis. Another study of 665 Chinese older adults (age between 60 to 95 years old) also using MoCA also found a negative correlation between sarcopenia and cognitive ability [45]. Different from the previous study, linear regression was applied and showed that low handgrip strength was associated with worse global cognitive function [45]. The present study also presents a positive correlation (β = 0.243, *p* < 0.001). The underlying pathophysiology for this relationship could be explained by adverse effects of chronic inflammation, impaired hypothalamic-pituitary axis, poor energy metabolism and oxidative stress [46].

### Relationships between FPG and CFA score

The relationship between glucose level and cognitive function remains controversial. In the present study, FPG level was found to be negatively correlated with CFA score in simple correlation, which corresponds with the finding of Yau et al. that older T2D patients with poor glucose control had better functional outcomes. They concluded that, in this age group, glucose control should not be too strict [47]. However, other studies have published opposite findings. Using the same MoCA measurement, Shimoda et al. found that diabetes patients had were more likely to have a MoCA score ≤ 25 (3.2) [48]. However, they did not use linear regression which could quantify the effects of glucose on the MoCA. Zaslavsky et al., studied in 316 participants over the age of 80, also confirming a positive correlation between glucose control and cognitive function (odds ratio, 0.18 points lower). However, this relationship attenuates in older groups. From their results, we might conclude that age plays a role in this relationship, which supports the findings of Yau et al. In the present study, the relationship between FPG and CFA was not significant in simple correlation. However, using Mach-L, FPG was identified as the last important factor to affect CFA. As mentioned in the methods section, the errors were all smaller in all four Mach-L, thus we suggest that Mach-L results are more reliable. Future studies with larger samples and longer time of follow-up are needed.

### Relationships between body fat, BMI and CFA score

It is interesting to note that both body fat percentage and BMI are the 5th and 6th important risks for low CFA score in T2D patients. This indicates that BMI and body fat are two independent factors and have different impacts on the pathophysiology of low CFA scores. It should be noted that body fat is the ‘genius’ fat composition of the human body. However, measuring body fat requires specialized equipment, whereas BMI is more easily obtained and is only an ‘estimation’ of human body fat based on body weight and height. This presents a significant drawback for BMI. For instance while bodybuilders have high body weight, most of their body composition is lean body mass. Waist circumference is another important indicator for body fat since it can be regarded as reflecting abdominal visceral fat which is more relevant to actual body fat. This is supported by Flegal et al., who found that WC and BMI are significantly more closely correlated with each other than with percentage body fat (*P* < 0.0001 for all sex-age groups [49]. Percentage body fat tends to be significantly more correlated with WC than with BMI in men but significantly more correlated with BMI than with WC in women (*P* < 0.0001). West et al., presented solid evidence for the role of body fat on cognitive function, finding that higher waist circumference was associated with future dementia after 8 year follow-up [50]. At the same time, directly measuring body fat with dual-energy x-ray absorptiometry, the Cardiovascular Health Study-Cognition Study found that higher body fat in men was significantly associated with increased dementia but only marginally associated in women in a cohort of 344 (non-diabetic) participants [51].

As for BMI, its relationship is opposite to that of body fat. Hu et al., followed 44,660 American T2D patients for 3.9 years, finding that higher BMI is associated with lower risk for dementia compared with normal BMI (< 25 kg/m^2^) [52]. A study in Korea also reached the same conclusion that all-cause dementia risk is lower in people with higher BMI (18.5—23 kg/m^2^) in T2D patients over the age of 40. The most generally accepted explanation for this correlation is that underweight is commonly associated with poor nutritional status which might result from the poor food intake and digestion [53]. However, the contradictory findings between BMI and body fat require further study with larger cohorts and more precise methods.

The present study is the first to re-evaluates the common risk factors of dementia, particularly in T2D patients using Mach-L approaches. While Mach-L has been criticized for its lack of operational transparency, it still effectively captures non-linear relationships between variables, making it highly useful for medical research. In the future, the use of multivariate adaptive regression splines could potentially provide greater operational insight and visualization.

Despite the improved understanding of the relative weights of risk factors for CFA score provided by Mach-L methods, the present study is still subject to certain limitations. First, the study is based on a relatively small sample, and further studies are needed with larger populations. Second, cross-sectional studies are less persuasive than longitudinal ones, and follow-up with T2D patients over a longer period will supply more information about the impact of these risks on CFA score. Thirdly, the methods used in the present study might be difficult or challengeable to other study group. However, the six most important impact factors identified are reasonable and consistent with previous findings. Lastly, while our study included the Montreal Cognitive Assessment, some participants opted out of the assessment for various reasons, potentially resulting in selection bias, thus caution must be taken when interpretating our results.

In conclusion, the four Mach-L methods could outperform MLR in our present study. Education level, age, frailty score, FPG, body fat, and BMI, were found to the be most important factors related to CFA in an older Chinese T2D cohort. Further study with a longitudinal design is warranted.

## Data Availability

The datasets used and/or analyzed for the current study are available from the corresponding author on reasonable request.
